# New York Medical Journal Association

**Published:** 1867-02

**Authors:** 


### NEW YORK MEDICAL JOURNAL ASSOCIATION.

Stated Reunion, January 11, 1867, Dr. Gurdon Buck in the Chair.

### PRESENT POSITION OF AURAL SURGERY.

Dr. Roosa remarked that the present method of examining the membrana tympani was a most decided improvement upon the somewhat familiar routine of the old practitioners. This revolution began with Prof. Troltsch's suggestion of the concave mirror, of six-inch focus and having a small central aperture for the convenience of the observer. Prof. T. combined with this the tubular speculum. There were those who even believed that the simple mirror would supersede Desormeaux's endoscope.

Another grand advance had been made in our appreciation of the real objects of interest, which present themselves for inspection. Formerly only the handle of the malleus and the periphery of the drum challenged attention. Now the triangular light spot, which effect, as first shown by Politzer, is due to the traction of the handle of the malleus upon the drum in an inward direction, was taking its proper rank in professional estimation. For the benefit of those who had given but little attention to aural science, he would pass around the illustrations of Dr. Politzer's work, for here this spot was prominently brought forward in the different phases of health and disease. He would also accompany those plates with certain photographic illustrations, obtained from Munich, in which the ossicula auditus and other parts of the ear were very well represented. The last number of Guy's Hospital Reports, as may be seen by a reference to the shelves of the Association, likewise exhibits perforation of the drum, although he objected to the term *normal* drum, as applied to one taken from the dead subject.

Then, again, the eustachian catheter, nowithstanding the assertion that Mr. Turnbull, in London, had killed two patients by its use, was steadily growing in the confidence of surgeons. Apropos of the danger to be encountered, he would answer with the experience of Continental and American surgeons, by whom this most excellent means of diagnosis was highly lauded. The only objection of value, and this a probable accident merely, was a rupture of the mucous membrane, which might produce an emphysema transient in character. Nothing alarming, so far, had occurred in his hands to induce him to abandon the instrument, unless a case of fainting might be regarded as an exception. Certain it is that the catheterism of the eustachian tube is an operation frequently performed in the Eye and Ear Infirmary, and in private practice, without any feeling of dread. The value of the procedure is demonstrable in chronic catarrhal inflammation of the middle ear (the chronic myringitis of Sir William Wilde), but it is not applicable to the treatment of a very rare affection, to wit, the stricture of the tube.

Bougies, for the strictures above alluded to, were now likewise benefically used. In this mode of treatment, Dr. Francis Simrock, of this city, had a large experience.

With regard to Politzer's method of inflation too much cannot be said. The late Mr. Toynbee proved that the eustachian tube was closed except during the action of certain muscles concerned in deglution; Politzer practicalized the discovery by requiring the patient to swallow, while the surgeon forced air through a tube, placed in one nostril while the other was closed. In this way, the rush of air through a ruptured membrane was readily appreciated, and at once became a valuable diagnostic sign. If during the experiment the membrana tympani chanced to be ruptured, there need be no apprehensions, since the wound thus caused very readily healed. Dr. R. spoke warmly of this method in those subacute cases, of which the following was a type, and which he would relate as exhibiting its success: A boy of scrofulous constitution, exposed to the action of cold, "gets," in common parlance, "his ears stopped up." This deafness promising to be permanent, he is treated according to the best intelligence of the period, by cauterization of the throat and removal of the tonsils, but without benefit. His physician, as soon as Politzer's method was promulgated, sent for the boy, applied the principle, and was rewarded by the fact that the patient heard an ordinary conversation in five minutes. This patient was again attacked a number of months after, and again in like manner relieved.

Politzer's method, however, was not valuable in chronic peripheral thickening, as shown by change in form of the light spot, etc. Its good effect was chiefly manifest in the removal of mucous accumulations which might take on structural changes. In perforations, Politzer's apparatus had been made available in thoroughly cleansing the canal established as a result of inflammation. This was accomplished by filling the affected ear with warm water, afterwards stuffing the meatus with raw cotton. The tube, cavity of tympanum and external meatus, were thus thoroughly washed out.

The nebulizer was now used in the therapeutics of aural diseases. Dr. Bishop's apparatus for nebulizing the mouth of the eustachian tube very often sends fluid instead of spray, but this freak sometimes being of advantage could hardly be urged as an objection. For the injection of iodine he had found Buttles's inhaler a very useful addition to Politzer's apparatus. Dr. R. alluded to and exhibited a few other appliances, upon the claims of which he descanted at some length.

Artificial membrane tympani were now found more generally applicable than before supposed. Mr. Toynbee's disc of rubber being liable to separate from its attachment and act as a foreign body in the ear, has been modified by a German, who has ended off the little wire with a spiral arrangement. The disc is secured by one or two of these closely fitting coils, in the same manner that a cork is by the cork-screw. In one case in Dr. R.'s experience the hearing distance was prolonged, during the use of these dics, from one or two inches to two feet, which was certainly a very comfortable gain to the patient.

Then, again, the otoscope, for listening to the passing of air into the middle ear, is a refinement in our means of diagnosis unknown to our predecessors. We have now also better defined ideas regarding the aftergrowths in the ear. What Mr. Toynbee was pleased to call exostoses of arthritic or syphilitic origin, are now accepted as being due to a primary periostitis. We expect, therefore, to prevent their formation. As an earnest, indeed, of the benefits to be derived from the future cultivation of this inviting field of inquiry, it is observed that aural polypi, which are now regarded as exuberant granulations merely, are much less frequently met with than they were ten years ago.

The change in nomenclature points out more exact knowledge of aural diseases, since such terms as otorrhœa, myringitis, etc., which only mislead the student are falling into disuse. We now know that the ceruminous secretions and the inflammation of the middle ear are independent of each other.

The *literature* of the subject is now no longer meagre; publications of high authority are readily attainable in London, Paris, Vienna, Munich, and New York. In addition to these facilities, we have a German quarterly devoted to the consideration of the topic. In fact, careful, earnest students are pursuing the branch with enthusiasm, and have already been rewarded by the most brillant returns. For the matter of that, statistics show that, exclusive of the chronic cases inherited by us from a previous generation, we are not more unsuccessful in our therapeutics here than in other parts of the body.

Dr. R., in the course of his remarks, gave some very interesting statistics, collected by himself and Dr. G. M. Beard in the asylums of New York and Hartford, the object of which was to fix the relation sustained by the pharyngeal mucous membrane towards the membrana tympani in the causation of congenital deafmutism. These, he promised, should be published in due time. He also maintained that the difficulties attendant upon the study of the disease were not as great as generally supposed—they need not interfere with the duties of the most active practice.

Dr. Ellsberg called attention to the omission on the part of

Dr. Roosa to mention the importance of inspecting the post-pharyngeal space. The element in diagnosis, in the course of Dr. R.'s extemporaneous remarks, had, no doubt, temporarily escaped the memory of the speaker.

Dr. Roosa acknowledged his indebtedness to the gentleman, and made a few remarks upon rhinosophy.

The Association then adjourned.

